# Ex vivo Dynamics of Human Glioblastoma Cells in a Microvasculature‐on‐a‐Chip System Correlates with Tumor Heterogeneity and Subtypes

**DOI:** 10.1002/advs.201801531

**Published:** 2019-02-10

**Authors:** Yang Xiao, Dongjoo Kim, Burak Dura, Kerou Zhang, Runchen Yan, Huamin Li, Edward Han, Joshua Ip, Pan Zou, Jun Liu, Ann Tai Chen, Alexander O. Vortmeyer, Jiangbing Zhou, Rong Fan

**Affiliations:** ^1^ Department of Biomedical Engineering Yale University New Haven CT 06520 USA; ^2^ School of Computer Science Carnegie Mellon University Pittsburgh PA 15213 USA; ^3^ Applied Math Program Yale University New Haven CT 06520 USA; ^4^ Department of Neurosurgery Yale School of Medicine New Haven CT 06520 USA; ^5^ Department of Pathology Indiana University Health Pathology Laboratory Indianapolis IN 46202 USA; ^6^ Yale Comprehensive Cancer Center New Haven CT 06520 USA

**Keywords:** brain tumor dynamics, ex vivo assays, microvasculature, organ‐on‐a‐chip

## Abstract

The perivascular niche (PVN) plays an essential role in brain tumor stem‐like cell (BTSC) fate control, tumor invasion, and therapeutic resistance. Here, a microvasculature‐on‐a‐chip system as a PVN model is used to evaluate the ex vivo dynamics of BTSCs from ten glioblastoma patients. BTSCs are found to preferentially localize in the perivascular zone, where they exhibit either the lowest motility, as in quiescent cells, or the highest motility, as in the invasive phenotype, with migration over long distance. These results indicate that PVN is a niche for BTSCs, while the microvascular tracks may serve as a path for tumor cell migration. The degree of colocalization between tumor cells and microvessels varies significantly across patients. To validate these results, single‐cell transcriptome sequencing (10 patients and 21 750 single cells in total) is performed to identify tumor cell subtypes. The colocalization coefficient is found to positively correlate with proneural (stem‐like) or mesenchymal (invasive) but not classical (proliferative) tumor cells. Furthermore, a gene signature profile including PDGFRA correlates strongly with the “homing” of tumor cells to the PVN. These findings demonstrate that the model can recapitulate in vivo tumor cell dynamics and heterogeneity, representing a new route to study patient‐specific tumor cell functions.

## 1 Introduction

The brain tumor perivascular niche (PVN), the region in the vicinity of microvessels, is a prime location for brain tumor stem‐like cells (BTSCs).^1^, ^2^ Tumor microvasculature creates a complex microenvironment consisting of various cell types, the extracellular matrix, and soluble factors that mediate cell–cell interaction.^3^ The brain tumor PVN controls maintenance, expansion, and differentiation of BTSCs via direct cell contact or paracrine signaling cues.^4^, ^5^, ^6^ BTSCs often receive bidirectional crosstalk from endothelial cells and other cell types in the niche.^7^ In addition, the perivascular zone may serve as a path for tumor cells to migrate over long distances.^8^, ^9^ Unlike other solid tumors, glioblastoma multiforme (GBM) cells rarely metastasize to other organs, but they can invade the entire brain by migrating along specific brain tissue structures, such as blood vessels or white matter tracts, leading to high rates of relapse.^9^, ^10^, ^11^, ^12^, ^13^ Despite the success in modeling diffuse brain tumors in both genetically modified and patient‐derived xenograft (PDX) animals,^14^ there is an unmet need for an in vitro system that can bridge conventional cell culture and animal models by mimicking not only the anatomy but also the function of the PVN to study the dynamics of BTSCs.

Traditional 2D cell cultures are incapable of replicating in vivo 3D environments where cancer cells reside, which may result in inaccurate data to evaluate drug responses.^15^, ^16^ Thus, there have been substantial efforts to develop 3D cell culture models, as well as patient‐derived tumoroids, that exhibit features closer to in vivo conditions.^17^, ^18^ These include areas of hypoxia, heterogeneous environment (e.g., stromal cells), different cell proliferation zones (quiescent vs replicating), extracellular matrix (ECM) influences, soluble signal gradients, and differential nutrient and metabolic waste transport.^19^, ^20^, ^21^, ^22^ Existing techniques used to culture cells into 3D structures include scaffold‐based approaches (e.g., polymeric hard scaffolds, biologic scaffolds, micropatterned surfaces)^23^, ^24^, ^25^ and non‐scaffold‐based approaches (e.g., hanging drop microplates, spheroid microplates containing ultralow attachment (ULA) coating, and microfluidic 3D culture).^22^, ^26^, ^27^ To date, current in vitro cancer models lack perfusable microvasculature, and thus, may not capture the essential role of microvascular niches in tumor progression and therapeutic response.^28^, ^29^, ^30^

Recently, perfusable microvasculature in microfluidic systems have been developed.^31^, ^32^, ^33^, ^34^, ^35^, ^36^, ^37^, ^38^, ^39^, ^40^ Several studies reported a spontaneous microvasculature formation via a vasculogenesis‐like process in a hydrogel loaded microfluidic chamber,^31^, ^32^ in which the seeding of endothelial cells in hydrogel‐loaded microfluidic networks leads to endothelial proliferation and lumen formation.^33^, ^34^, ^35^, ^36^ Kamm et al. developed a microfluidic device containing 3D perfusable microvessel networks to investigate tumor cell intra‐ and extravasation.^37^, ^38^ Phan et al. reported that 3D microtumors/tumoroids made of colorectal, breast, and melanoma tumor cell lines can be revascularized in a perfusable diamond‐shaped microchambers to study drug responses.^41^ To our knowledge, current vascularized‐tumor‐on‐a‐chip models have incorporated cancer cell lines as a proof‐of‐concept, but it has not yet been demonstrated that such a platform can work as a pathophysiologically relevant system to evaluate the function of primary tumor cells ex vivo in a patient‐specific manner.

We report on the first demonstration that a tissue‐engineered microvasculature‐on‐a‐chip system can be utilized to examine the function of primary patient‐derived BTSCs. Live cell imaging of tumor cell dynamics and localization allowed for delineating the interaction of single brain tumor cells with the nearby microvessels. We found that BTSCs preferentially localize in the region within ≈50 µm from microvessels and that a fraction of these cells directly attach onto the vessels. We further validated our results using single‐cell RNA sequencing of 10 GBM patients (26 batches, 21 750 single cells). We found that tumor‐microvessel colocalization was based on the genetic and pathologic subtypes of the tumor samples. Our GBM‐microvasculature‐on‐a‐chip model demonstrates the potential for ex vivo analysis of tumor cell functions and patient‐specific GBM treatment.

## 2 Results and Discussion

### 2.1 Perfusable Endothelialized Microvasculature‐on‐a‐Chip

**Figure 1:**
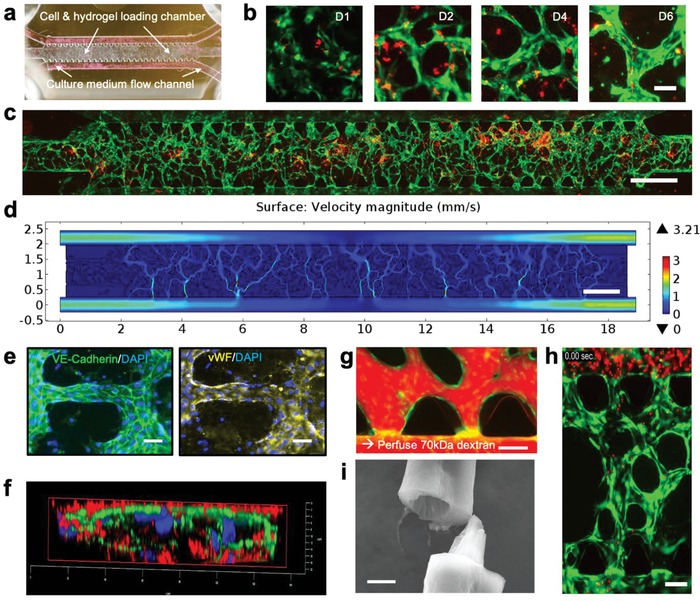
Growth of BTSC‐incorporated microvasculature‐on‐a‐chip. a) Microfluidic device (250 µm in height) containing a cell/gel loading microchamber (8000 µm × 1300 µm) flanked by two medium flow channels (500 µm in width). An array of triangular microposts separate the gel chamber and the medium flow channel, allowing for loading and confining the hydrogel precursor to the midchamber only. Scale bar: 1000 µm. b) Representative time course images of the microvessel formation over a period of 6 d. Scale bar: 15 µm. Green: GFP‐HUVEC. c) Whole chip scan showing microvasculature formation (96 h postcell in fibrin) and loading of single BTSCs (GS5). Green: GFP‐HUVECs. Red: BTSCs. Scale bar: 1000 µm. d) Comsol Finite Element Simulation of flow velocity magnitude (mm s^−1^). The finite element model was constructed using the experimental whole‐chip microvessel network in (c). e) Immunostaining of VE‐cadherin and vWF to examine the formation of adherens junction and the function of tissue‐engineered endothelial vessels at day 3. Scale Bar: 20 µm. f) Cross‐sectional confocal image showing two adjacent microvessels and collagen IV deposition. Green: GFP‐HUVECs. Red: collagen IV. g) Infusion of 70 kDa fluorescent dextran to measure impermeability of the lumen and examine microvessel opening to the media channel (anastomosis). Green: GFP‐HUVECs. Red: Dextran. Scale Bar: 110 µm. h) Flowing fluorescent microbeads (red) (10um) through a microvessel network (green) that were grown for 3 d. Scale Bar: 100 µm. i) An SEM image of the microvessel. Scale Bar: 5 µm.

Our microfluidic device (AIM Biotech) is comprised of a center chamber for loading a mixture of endothelial cells and hydrogel precursor and two lateral channels for perfusing culture medium (**Figure** **1**a). The cell/hydrogel loading chamber and two lateral channels are separated by triangular microposts which prevent gel leaking.^42^ Human umbilical vein endothelial cells engineered for expression of green fluorescence protein (GFP‐HUVECs, Angio‐Proteomie) were suspended in a 2.5 mg mL^−1^ fibrin gel precursor and loaded into the microfluidic chamber. Premature nascent lumens developed in 3 d via a vasculogenesis‐like self‐assembly process. A connected microvessel network spanning the entire chamber (≈1.3 mm × 8 mm) was established in 4–6 d (Figure S1a,b, Supporting Information) upon daily perfusion with medium. Previous studies showed that adding stromal cells (e.g., mesenchymal stem cells and lung fibroblasts) which secrete proangiogenic factors in the hydrogel accelerated vasculogenesis and expedite the formation of interconnected perusable microvascular network in as little as 3 d.^43^ However, because such stromal cells are not naturally found in the brain niche, we did not include them in our model. Instead, we used medium containing a cocktail of growth factors (vascular endothelial growth factor (VEGF, 50 ng mL^–1^), fibroblast growth factor (FGF, 20 ng mL^‐1^), epidermal growth factor (EGF, 20 ng mL^‐1^)) to promote in vitro microvasculature growth. Anastomosis of microvessels was observed at the vessel openings to medium flow channels, which allowed for perfusion and the establishment of shear stress in the microvascular network to foster microvessel development and maturation.

### 2.2 Incorporating Primary GBM Cells in the Microvasculature‐on‐a‐Chip System

To investigate the behavior of BTSCs in the microvascular environment, we used a well‐characterized, patient‐derived brain tumor neurosphere culture, GS5 (Figure S2a,b, Supporting Information). Unlike U87 cells, GS5 cells are enriched for tumor stem‐like cells and can generate highly infiltrative tumors in mice, mimicking human GBM histopathologically.^29^, ^44^ Compared to U87‐derived tumors, GS5‐derived tumors more accurately recapitulate the infiltrative nature of GBM in xenograft mouse models.^44^ In addition, GS5 cells exhibit a stem‐like phenotype with expression of multiple neurodevelopmental genes. As an intermediary between U87 cells, a stable cell line that loses many important GBM features, and primary patient‐derived tumor cells, GS5 cells served as a reference for patient‐derived stem‐like GBM cells. GS5 cells were mixed with GFP‐HUVECs in the hydrogel precursor at a 1:4 ratio and loaded into the microfluidic device to grow 3D microvasculature. Endothelial cells began to connect together to form networks by day 2 and grew into extensive microvessels by day 4 (Figure 1b). Tumor cells exhibited motility while the microvessels were remodeling until reaching a relatively stable geometry by day 6.

A whole chip scan (Figure 1c) showed the formation of interconnected microvessel network in day 4. After determining the patency of the microvessel network, finite element simulations were performed to determine the local flow rates (Figure 1d) and shear stresses in the microvessel bed. Florescent images were vectorized and imported into COMSOL Multiphysics to simulate the flow of media. With a gradient of 10 Pa, the maximum shear stress experienced by microvessels was 4.02 dyne cm^−2^ and the maximum flow rate in the microvessels was 3.21 mm s^−1^. The simulations revealed the heterogeneity that exists within the microvessels and how flow and shear properties influence the interaction between tumor cells and the adjacent vessels.

To confirm that tissue‐engineered microvessels are physiologically functional and resemble native microvessels, we examined the formation of lumen and the presence of protein markers indicative of essential physiological characteristics. Confocal imaging showed that our microvessel network developed open hollow lumens as early as day 3 (Figure S1c, Supporting Information). Immunostaining of the fixed microvessels for VE‐Cadherin revealed the formation of endothelial cell adherens junction over a large area of the microvascular network (Figure 1d, left). In response to shear stress induced by medium perfusion, microvessels produced von Willebrand factor (vWF), a secreted factor that was deposited onto the inner wall of endothelia and polymerized to form vWF fibers that can facilitate platelet adhesion (Figure 1d, right). Cross‐sectional confocal imaging also revealed that endothelial cells secreted and deposited collagen IV to the basal surface of lumen, forming a 3D collagen IV mesh as early as day 3. This collagen layer not only stabilizes microvessels but also constitutes an important ECM component of the PVN in our model (Figure 1f). This also demonstrated endothelial cell polarization during lumen formation. In addition, the production of collagen IV and deposition of ECM are indicative of microvessel maturation.

To further evaluate the quality of microvessels, we tested the permeability via the perfusion of 70 kDa fluorescently labeled dextran (Figure 1g). We used time‐lapse imaging to calculate a permeability coefficient of (1.91 ± 0.45) × 10^−6^ cm s^−1^ (*n* = 4, at day 6), which was comparable to previously reported results for in vitro microvessel models.^32^, ^43^, ^45^ This permeability coefficient is higher than that in the mural/stromal cell supported microvessels, suggesting that microvessels made of a mono‐layer of endothelial cells are leakier in the absence of other vascular mural cells, such as fibroblasts and pericytes. Furthermore, a suspension of 10 µm sized fluorescent polystyrene beads was perfused into the upper channel of a 3 d old chip. We observed that the beads readily traveled through the microvascular network and entered the lower microchannel with minimal adherence to the microvessel wall (Figure 1h and Movie S1, Supporting Information). Finally, the microvasculature hydrogel slab was retrieved, fixed, and dehydrated for scanning electron microscopy (SEM) to confirm the formation of 3D architecture of interconnected endothelial lumen network (Figure 1i).

### 2.3 Preferential Localization of BTSCs in PVN

**Figure 2:**
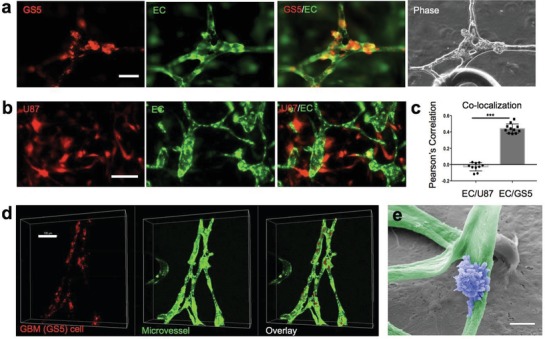
Quantification of tumor cell localization relative to microvessels. a) Phase and fluorescent images of microvessels with BTSC GS5. BTSCs were incubated with the Dil membrane dye for 40 min prior to coculture with GFP‐HUVECs. BSTCs localize more preferentially to the branching points of the microvessel network. Scale bar: 80 µm. b) Fluorescent images of GBM cell line RFP‐U87 cells loaded in a microvessel network. RFP‐U87 cells randomly distributed in the gel space and the microvessels constantly remodeled. Scale bar: 80 µm. c) Quantitative analysis of colocalization of GS5 versus U87 cells with the microvessels. d) Confocal images to examine colocalization of GS5 cells and microvessels. Red: GS5 cells. Green: HUVECs. Scale Bar: 100 µm. e) SEM image (false color) showing a BTSC on the microvessel. Scale:5 µm.

The role of PVN in controlling BTSC fate has been reported in human GBM and validated with animal xenograft models.^4^, ^5^, ^46^, ^47^ Using tissue‐engineered microvasculature models to determine whether BTSCs preferentially localize within the PVN, we quantified colocalization of microvessels and BTSCs (GS5) relative to a GBM cell line (U87). Tumor cells were prestained with lipophilic cell tracking dye Dil (Invitrogen), mixed with GFP‐HUVECs, and loaded into the microfluidic chip to examine microvessel growth and tumor cell dynamics. After 7 d, we observed that BTSCs preferentially localized in the perivascular zone (**Figure** **2**a), specifically in the bifurcation region of the microvessel network. In contrast, U87 cells were overpopulated and did not colocalize (Figure 2b). In addition, we observed that incorporation of U87 cells led to fast microvessel remodeling and unstable microvessel network formation, whereas GS5 cells resulted in well‐connected microvessel network in 4–5 d. Previous in vivo studies reported that U87 failed to accurately model human GBM compared to patient‐derived tumor stem cells.^30^, ^48^ Researchers characterized multiple GBM cell lines and showed that U87 exhibits high mitotic figures (as measured by Ki67) but low levels of neural stem cell markers, such as nestin, Sox2, and CD133.^48^, ^49^ Our result is concordant with previously reported studies that compare different cell sources for tumorigenic GBM models. In practice, pathologists diagnose GBM based on three golden standards: mitoses, microvascular proliferation, and necrosis.^50^ However, it is not fully characterized how proliferative GBM cells migrate and distribute in the brain relative to the vascular system.^51^ We observed that GS5‐EC coculture system exhibited a more connected vessel network. GS5 cells resided in the region near microvessels, whereas U87 showed a different localization pattern in a similar device. We used ImageJ to determine the colocalization of tumor and microvessel signals in the same image by quantifying the Pearson's correlation coefficient (see the Experimental Section). Quantitative analysis confirmed that patient derived BTSCs (GS5) showed a significantly higher Pearson's correlation coefficient (0.44± 0.02, *n* = 11) than that of U87 cells (−0.03 ± 0.02, *n* = 10) (Figure 2c) 7 d after loading into the microchip. One popular hypothesis of tumor nutrient supply is that tumor cells can respond to the existing blood vessels (vessel co‐option).^8^ As we could observe and evaluate the relative tumor‐vessel location, our model may serve as a high‐throughput platform to test anti‐vessel‐co‐option drug in vitro.

Scanning confocal microscopy was used to show the 3D location of BTSCs (red) relative to microvessels (green) (Figure 2d). The distribution of BTSCs in this 3D hydrogel slab mirrored the structure of the microvessel network. We did not observe tumor/endothelial cell colocalization where the perfusable lumens did not form with U87. Furthermore, about 30% of BTSCs appear to be fully integrated in the endothelia of microvessels (Figure 1c). It has been reported that brain tumor stem cells can differentiate into vascular cells, such as pericytes or endothelial cells, and contribute to tumor angiogenesis.^52^, ^53^, ^54^ We did not observe any tumor cell intravasation or transdifferentiation across vessel wall on our chips. We found that most tumor cells adhere and spread onto the surface of microvessels, presumably via adhesion to collagen IV mesh produced by endothelial cells (Figure 1e). In contrast to the smooth surface of endothelialized microvessels (Figure S2c,d, Supporting Information), cell surfaces of BTSCs are very rough and decorated with extensive vesicles (Figure S2e, Supporting Information), suggesting their secretory activity to elicit a more complex cell–cell communication network in the PVN.^55^, ^56^ Recent studies report that secreted extracellular vesicles of BTSCs contain proangiogenic factors (e.g., VEGF) that may contribute to vessel formation.^55^, ^57^ This may explain why the chip containing GS5 cells and HUVECs exhibited a more branched microvessel network, while the chip containing U87 and HUVECs barely formed any perfusable vessels.

Our findings indicate the potential of a tissue‐engineered microvasculature‐on‐chip system as a functional surrogate to examine the interaction between tumor cells and the PVN, and potentially as a platform for ex vivo assay of tumor cell properties. Although it is unclear where and how tumor cells decide to reside in the niche, we propose they are related to the signals associated with oxygen gradients, nutrient gradients, endothelial cell‐secreted factors, and interactions between BTSCs and ECM on the vessel surface.

### 2.4 Tracking Tumor Cell Migration in PVN

**Figure 3:**
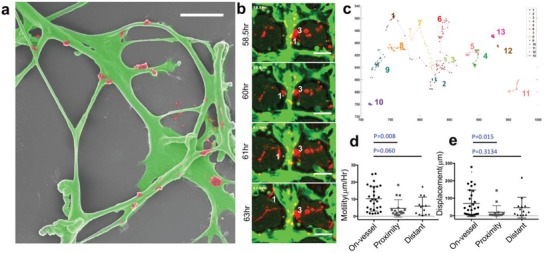
Tumor cell migration in the microvasculature‐on‐a‐chip. a) SEM image (false color) of a typical microvessel network (green) with GS5 cells (red). Scale: 50 µm. b) Fluorescence images of a representative region at different time points to track tumor cell migration. c) Migration trajectory of 13 tumor cells in the region shown in (b) for a day 3 microchip and measured over a period of 20 h. Axes unit: pixel. d) Average motility (µm h^−1^) of single tumor cells from three groups defined by the initial location of tumor cells relative to microvessels. On‐Vessel (10.29 ± 7.23 µm, *n* = 28); Proximity (4.60 ± 5.11 µm h^−1^, *n* = 16); distant (5.94± 5.29 µm h^−1^, *n* = 13). ANOVA test (*p* = 0.0133, Kruskal‐Wallis Test). e) Absolute displacement of single tumor cells from three groups. On‐Vessel (70.71 ± 75.96 µm, *n* = 28); proximity (19.32 ± 38.42 µm, *n* = 16); distant (46.28± 59.5 µm, *n* = 13). ANOVA test (*p* = 0.0042, Kruskal‐Wallis Test).

We observed extensive colocalization of GS5 BTSCs in the perivascular region 7 d after the formation of interconnected microvessel network (**Figure** **3**a). However, how each cancer cell migrates relatively to microvessels to eventually “home” in the perivascular region remains unclear. To explore the underlying mechanisms, we utilized live cell tracking fluorescence microscopy (Figure 3b) to image GS5 cells in the microvasculature‐on‐a‐chip device 2 d after cell seeding for a period of 20 h (48–68 h post‐cell loading) at a rate of two scans per hour. We observed that most tumor cells (i.e., cell 3 in Figure 3b) residing in the perivascular region had reduced migration rates and a round morphology. The cells more distant from the microvessels showed spindle‐like morphology and extended filopodia but did not migrate over long distances. Surprisingly, the most migratory cells (i.e., cell 1 in Figure 3b,c) were located very close to or traveled along the microvessels (Figure S3b, Supporting Information). It is known that brain tumor cells utilize the microvascular tracks to invade distant regions of the brain. The migratory phenotype is predominately de‐differentiated or mesenchymal, resembling the GBM mesenchymal subtype that often causes nonresectable disease.^58^, ^59^, ^60^ Our result is consistent with prior in vivo studies^12^ and suggests that our set‐up is potentially capable of differentiating between highly invasive tumor cells and stem‐like quiescent tumor cells.

Next, we calculated cell motility and migration distance to determine the cell migration trajectory (Figure 3c, Figure S3a and Movie S3, Supporting Information). We grouped the cells based upon each cell's relative distance to the nearest microvessel into three categories: *On‐Vessel* (the distance to vessel in our fluorescence image = 0), *Proximity* (the distance to vessel < 50 um) and *Distant* (the distance to vessel > = 50 um which is considered as outside the PVN). Statistical analysis (one‐way ANOVA) demonstrated significant differences in the total displacement (*p* = 0.0121) or motility (*p* = 0.0134) (Figure 3d,e) among these groups. Tumor cells residing in the PVN were mostly round but the ones outside the PVN were constantly extending filopodia to explore their surroundings. Interestingly, the lowest migratory distance cell group was not *On‐Vessel* but the *Proximity* group. The *On‐Vessel* group was up to 5x higher motility compared to *Proximity* group. We hypothesized that the direct interaction between tumor cells and the collagen mesh on the vessel surface was responsible for facilitating tumor cell adhesion and directioning migration.

The surface marker phenotype was also measured by immunostaining for nestin and Sox2, and the result confirmed that a significant fraction of BTSCs was perivascular. In the PVN, 61.6% ± 2.2% (*n* = 3, day 7) of BTSCs were nestin‐positive cells and 68.2 ± 4.0% positive for Sox2. The observation of tumor cell differential dynamics in PVN was unanticipated and demonstrated the feasibility to use live cell dynamics measured in our device to assay the functional phenotype of single brain tumor cells in a physiologically relevant environment.

### 2.5 All Patient Samples: Colocalization of Tumor Cells and Microvessels

**Figure 4:**
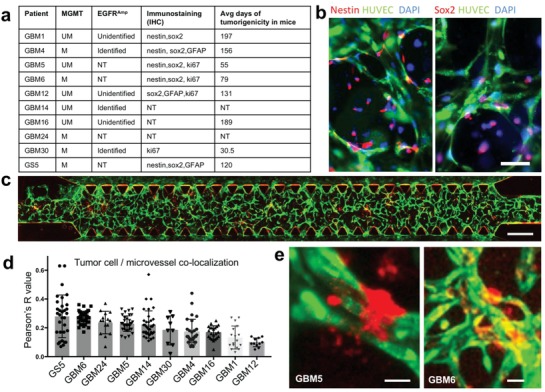
All patient samples: tumor cell localization relative to microvessels. a) Patient information table. All GBM samples are IDH wild‐type. M, MGMT promoter methylated; UM, MGMT promoter not methylated; NT, not tested. b) Immunostaining of nestin and Sox2. Scale: 30 µm. c) Whole chip scan of GBM6 in the microvasculature chip at Day 4. Scale: 700 µm. Red: GBM6 cells. Green: HUVECs. d) Colocalization coefficient of tumor cells and microvessels measured for all patient samples. e) Representative images of patient cells in the microvasculature chip. Red: GBM cells. Green: HUVECs. Scale: 10 µm.

To assess interpatient heterogeneity of brain tumor cells' PVN “homing” ability, we applied the same approaches to evaluate additional 9 patients' BTSCs. All patient samples (**Figure** **4**a) used were IDH wild‐type, GBM, with differing O^6^‐methylguanine–DNA methyltransferase (MGMT) promoter methylation, epidermal growth factor receptor (EGFR) amplification status, stem‐cell markers Sox2 or nestin, (Figure 4b), and ability to grow tumors in mice. A representative whole chip scan (sample GBM6, Figure 4c) showed extensive microvessel growth by day 4, while BTSCs largely remain isolated. The relative distance between each tumor cell and the nearest microvessel was measured to calculate a Pearson's colocalization coefficient R value for each patient sample. The results together with GS5 cell data in day 4 were rank ordered and plotted in Figure 4d. GS5 ranked highest, and other top ranked patient samples included GBM6, GBM24, and GBM5. The lowest colocalization coefficient in the group was patient GBM12, with a colocalization coefficient nearly a third of that for GBM6. We noticed significant morphological differences in tumor cells when interacting with microvessels (Figure 4e). For example, the typical perivascular GBM5 cells wrapped around the microvessels and actively produced microvesicles. The morphology of GBM6 cells in the microvessel network resembled that of brain tumor microvasculature in murine.^12^ Finally, we observed an association between colocalization and poor prognosis in the xenograft. For example, GBM5 and GBM6 had relatively high colocalization coefficients and rapid tumor growth (<100 d), while the four samples ranked lowest in the localization coefficient (include which samples these are) had the best prognosis after tumor formation in xenograft mouse models (>100 d). This points to a potential application of our ex vivo on‐chip tumor cell localization assay to predict both in vivo and clinical outcome.

### 2.6 Single‐Cell RNA‐Seq to Correlate with Transcriptional Subtypes and Gene Signatures

**Figure 5:**
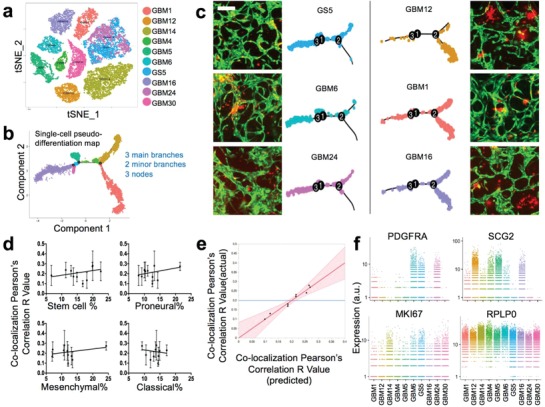
Single‐cell RNA‐seq correlates on‐chip colocalization to transcriptional signatures/subtypes. a) tSNE plot of single cell RNA‐seq data from all patient samples. b) Single‐cell pseudotime lineage trajectory obtained by semisupervised clustering of subtype‐specific gene panel using Monocle. c) Representative images of tumor cells in microvasculature and the matched Monocle plots for top three and bottom three colocalization coefficient samples (Day 4–7). Scale bar: 200 µm. d) The linear regression model showing the percentage of GBM subtype in each sample in correlation with the colocalization R value. e) The multivariate linear model showing the predictor variables (average gene expression of PDGFRA, C1GALT1, THY1, and MKI67) as a combined transcriptional signature that correlates with colocalization. f) Relative gene expression of angiogenesis (PDGFRA, SCG2), proliferation (MKI67), and housekeeping marker (RPLP0) in single cells from all patient samples.

To correlate the observed brain tumor cell behavior heterogeneity with the microvasculature‐on‐a‐chip system to tumor cell genotype or phenotypes, we conducted single‐cell 3' mRNA sequencing of all ten patient samples (Table S1, Supporting Information) and obtained 26 027 single‐cell transcriptomes at a depth of at least 10 000 reads per cell. To minimize the sequencing batch‐to‐batch bias, we prepared 2–3 batches of cDNA libraries for each patient sample for a total of 26 batches. The mean of transcripts (UMIs) per cell detected in each batch ranged from 6192 to 20174. The median of genes detected per cell ranged from 1740 to 3626. In total, 24 120 genes in 21 750 cells passed the Seurat quality control filtering (see the Experimental Section) and were used for downstream analysis (Table S1, Supporting Information). The whole transcriptome of all single cells was used to perform differential gene expression and clustering analysis. The result was analyzed by both Principal Component Analysis (PCA) (Figure S5a–c, Supporting Information) and t‐distributed stochastic neighbor embedding (tSNE) (**Figure** **5**a, Figure S5c–d, Supporting Information). Unsupervised tSNE clustering based on the top 1000 highly variable genes suggested that interpatient heterogeneity was stronger than the global transcriptional state of between tumor cells within the same sample, which was consistent with a previous report.^61^

To identify the transcriptional programs intrinsic to GBM cells and compare them across all ten patients such that the observed ex vivo behaviors in the PVN microchip model can be associated with molecular mechanisms, we performed the following informatics analysis. First, we did single‐sample Gene Set Enrichment Analysis (ssGSEA)^62^ with the gene signatures previously reported^63^ to quantify tumor cells expressing stem cell characteristics and found that most patient samples had a high percentage (12.05–17.49%) of stem‐like cells, except for GBM24 (8.77%) and GBM30 (5.79%) (Table S2, Supporting Information). Second, we validated single tumor cell subtypes compared to a permutated data set (permutation = 1000) in ssGSEA. According to The Cancer Genome Atlas (TCGA) data published for human GBM, there are four major genotypes: classical, mesenchymal, proneural, and neuronal, each of which has specific transcriptional signatures (684 genes in total).^58^ However, at the single‐cell level, a patient's tumor could consist of multiple subtypes, with the leading subtype presumably defining the genotype of bulk tumor.^63^ At the transcriptional level, a few tumor cells may be enriched for gene markers of two subtypes. Therefore, we defined the subtype of a tumor cell as the dominant gene set with p‐value<0.2 (Table S3, Supporting Information). Recent studies suggested the removal of the neuronal subtype due to its overlap with the proneural subtype and suboptimum identification using current gene signatures.^62^, ^63^ In order to further visualize the lineage relationship between tumor cells in each sample, we applied a pseudotime differentiation trajectory method to analyze all single cells using the gene sets associated with three major subtypes—classical, mesenchymal, and proneural. We then reconstructed the pseudotime lineage relationships with three subtype markers (555 genes) using semi‐supervised analysis in Monocle package.^64^, ^65^ The resulting plot had three major branches and three major nodes connecting them with two minor branches (Figure 5b). The differential gene expression between branches was shown in Figure S6 (Supporting Information). Next, we investigated whether tumor/microvessel colocalization (Figure 4d) correlated with tumor cell heterogeneity and subtype. We found that the top three highly colocalized tumor samples (GS5, GBM6, and GBM24) shared a similar cellular trajectory, in sharp contrast with the graph of the three least colocalized tumor samples (GBM12, GBM1, and GBM16) (Figure 5c), indicating a strong correlation between tumor genomic subtypes and ex vivo tumor cell dynamics in the microvasculature‐on‐a‐chip system.

Linear regression analysis was performed to compare the percentage of stem‐like, classical, mesenchymal, or proneural GBM cells to the colocalization coefficient (Figure 5d). Although none of them reached statistical significance, we observed a slightly positive colocalization with the percentages of stem‐like, mesenchymal, and proneurnal cells, but not classical cells. This finding is in agreement with previous reports,^46^, ^66^ which studied the role of the PVN in both stem‐like cell fate maintenance and vascular track invasion and found mesenchymal cells to be the most invasive subtype. The BTSC PVN model was primarily validated with the proneural subtype,^67^, ^68^ but not the classical subtype, which has high probability of EGFR amplification and features enhanced cell proliferation and tumor growth. However, the entire panel of GBM subtype genes (684 genes) did not result in a statistically significant colocalization correlation. Furthermore, we examined specific gene markers with these panels but that were associated more with pro‐angiogenesis and the interaction with endothelium (Figure S7, Supporting Information). Based on a multivariate linear mixed model with patient level effect adjusted, we tested if the average gene expression level of marker genes related to the colocalization coefficient. We found four genes (PDGFRA, C1GALT1, THY1, and MKI67) were significantly associated with the colocalization coefficient (Figure 5e, Table S4, Supporting Information). Platelet‐derived growth factor receptor alpha (PDGFRA), one of the most distinct signature genes for proneural GBM, was found to be expressed highly in the top three ranked tumor cells. This finding further supports our hypothesis that the “homing” of BTSCs in the PVN is well demonstrated in proneural models but not others.^67^, ^68^, ^69^ Endothelial cells in the vessels usually recruit pericytes or mescenchymal cells via PDGF signaling towards vessel maturation.^70^ We propose that tumor cells with a high expression level of PDGFRA respond better to PDGF secreted by endothelial cells, and thus show a high vessel colocalization ratio. Although PDGF and PDGFRA are coexpressed in GBM, we found that amplified expression of PDGFRA, not PDGF nor PDGFRB, correlated with tumor cell‐vessel colocalization. Previous clinical trials using PDGF receptor antagonists have been disappointing.^71^, ^72^ This could be due to the interpatient and/or intrapatient heterogeneity of GBM cells, which is consistent with the mRNA data in our study (Figure S5d, Supporting Information). Recent advances in genetic profiling suggests that combination therapy targeting multiple receptor tyrosine kinases (RTKs) including PDGFRA, EGFR, and MET could provide better efficacy.^73^, ^74^ SCG2, a gene associated with secretory function, was found to negatively correlate with colocalization, except GBM5, which was confirmed as an active extracellular vesicle producer (Figure 4e). GBM30 was found to express low levels of both PDGFRA and SCG2, but showed the highest proliferation and tumor growth capability in mouse xenografts. VEGFA, one of the most important proangiogenic factors, did not show significant differences in most patient samples and no correlation with colocalization coefficients.

These results suggest that ex vivo behaviors of BTSCs in a 3D microvasculature model can recapitulate pathophysiological characteristics, as shown by our model of the PVN. Additionally, the ability to “home” to the PVN of single tumor cells can be associated with transcriptional subtype and correlates with interpatient heterogeneity. This is the first demonstration that a tissue‐engineered 3D microvasculature system can provide a functional niche to assay the dynamics of primary tumor cells derived from patients, opening a new direction for organ‐on‐a‐chip applications.

## 3 Experimental Section

*Cell Culture*: Primary culture of HUVECs were purchased from Yale Vascular Biology and Therapeutics Core. GFP‐HUVECs were commercially obtained (Angio‐Proteomie) and cultured in endothelial growth medium EGM‐2 (Lonza) with full supplements. HUVECs and GFP‐HUVECs between passage 2 and 6 were used in all experiments. No significant difference between HUVECs and GFP‐HUVECs in vessel formation ability was observed. Red fluorescent human GBM cells (RFP‐U87) and patient‐derived glioma stem‐like cells (GS5) were provided by Prof. Jiangbing Zhou's lab at Yale University. RFP‐U87 were cultured in Dulbecco's modified Eagle's medium (DMEM) supplemented with 10% fetal bovine serum (FBS). All GBM specimens were provided by Yale Neuropathology Service. Fresh patient‐derived GBM cells were isolated and cultured from GBM patient surgical specimens with approval from the Institutional Review Board at Yale‐New Haven Hospital. Extensively rinsed tumor specimens were finely minced and placed in DMEM/F‐12 medium (Gibco) with 25 unit mL^−1^ Papain (Worthington Biochemical Corp). A series of mechanical dissociations was used to obtain a single‐cell suspension. Resuspended cells were cultured in neural basal medium supplemented with B27 (Gibco), FGF (20 ng mL^−1^, Peprotech), and EGF (20 ng mL^−1^, Peprotech). Brain tumor‐derived neurospheres were evident as early as one week after plating.

*Chip Loading and Maintenance*: Microvessels formed by HUVECs were cocultured with glioma cells in a stack of fibrin gel in the 3D cell culture chips (AIM Biotech). The chip, adapted from Prof. Roger Kamm's design, consisted of three parallel channels: one central cell‐containing gel loading channel and two lateral medium flow channels.^75^ Endothelial cells were seeded in the fibrin gel by introducing HUVECs (2 × 10^6^ cells mL^−1^) and GBM cells (0.5 × 10^6^ cells mL^−1^) in the 2.5 mg mL^−1^ fibrinogen (Sigma) dissolved in serum‐free neural basal medium. Thrombin (5 U per 10 mg fibrinogen, Sigma) was then added to convert the soluble fibrinogen into insoluble fibrin strands. Immediately after gentle mixing, the gel (≈10 µL) was pipetted into the gel‐loading channel of 3D cell culture chips. The samples were placed in a humidified 5% CO_2_ 37 °C incubator for 40 min, to allow the fibrin to polymerize. 50% EGM‐2 media and 50% neural basal medium supplemented with B27, VEGF (50 ng mL^−1^), EGF (20 ng mL^−1^), and FGF (20 ng mL^−1^) was then added to the two lateral flow channels. The gravity‐driven flow was introduced by adding 50 µL of medium into two connected ports of the same channel and 70 µL into the opposite two connected ports. This differential volume created a transient flow across the porous fibrin gel and the anastomotic microvessels in the chip. Media was changed every 12 h for a period of up to 2 weeks.

*Immunofluorescent Staining and Imaging*: For live cell tacking, brain tumor stem‐like cells GS5 were incubated with Dil cell membrane dye (1:200, Invitrogen) for 40 min in a humidified 37 °C incubator. For immunofluorescent staining, devices were fixed by 4% paraformaldehyde (ChemCruz) for 20 min at room temperature. Primary antibodies were used at 1:100 overnight at 4 °C and secondary antibodies were used at 1:1000 for 1 h at room temperature (Table S6, Supporting Information). The 3D microvessels were imaged using a confocal microscope (Leica DMi8) and deconvoluted by Hyugens Professional software (Scientific Volume Imaging). Unless otherwise stated, all other images were taken with Nikon Eclipse Ti‐S microscope and processed with NIS‐Elements software (Version 4.20, Nikon Instruments).

*Single Cell 3' mRNA Sequencing*: Our approach for high‐throughput single cell mRNA sequencing was based on a closed microwell array chip developed in our lab.^76^ Microwell arrays were used as the platform for coisolating single cells and uniquely barcoded mRNA capture beads for single cell mRNA capture following lysis. The dimensions of microwells are dictated by the size of mRNA capture beads (≈35 µm); and chosen as ≈45–55 µm in diameter and ≈50 µm in height to ensure accommodation of only a single bead as well as most mammalian cell types. This choice of dimensions also facilitates straightforward removal of beads after mRNA capture either by purging (for closed‐environment cell seeding) or centrifugation (for open‐surface cell seeding) after inverting the devices. The throughput of the microwell arrays is up to 70 000 wells to be able to sequence ≈1000–5000 cells in a single run where the arrays are loaded with a well occupancy rate of 5–10% to minimize dual occupancy (cell duplets).

Master wafers for microwell arrays were fabricated using SU‐8 negative resist. A single layer of resist (SU‐8 2035, MicroChem) was spun at 2200–2400 rpm for 30 s to yield feature heights of ≈50 µm. The wafers were then exposed to ultraviolet light through a transparency mask (CAD/Art Services) to pattern microwells. After developing and baking, wafers were hard baked at 150 °C for 30 min, and silanized for 2 h in a vacuum chamber saturated with Trichloromethylsilane (Sigma‐Aldrich). Fabrication of microfluidic channels followed a similar fabrication procedure using SU‐8 2075 where channel height was set to ≈120 µm (1700–1800 rpm for 30 s).

Devices were made by casting polydimethylsiloxane (PDMS, Sylgard 184, Dow Corning) over the master wafers followed by degassing and curing at 80 °C for 6–8 h. Both microwell array and microfluidic channel device were set to a final height of 3–4 mm. After curing, PDMS was peeled off, and devices were cut to proper sizes to fit on a glass slide. For microfluidic channels, holes for fluidic connections were punctured using a biopsy punch (Miltex, 1.5 mm). Microwell arrays were first plasma‐bonded to microfluidic channel and then to a glass slide.

For single cell RNA‐seq experiments, the patient‐derived GBM neurospheres were cultured for 3–5 weeks in serum‐free media as described above and then gently pipetted with a 200ul pipette tip to dissociate into single cells. Dissociated single cells and mRNA capture beads were then inputted into the microwell chip sequentially using a pipette and allowed to settle into wells by gravity using syringe‐pump driven flow. Freeze‐thaw lysis buffer was introduced into the microchannel followed by fluorinated oil (Fluorinert FC‐40) to seal each microwell to prevent cross‐contamination. Cell lysis was achieved using three freeze‐thaw cycles, and cell lysate and beads were incubated at room temperature for 60 min for mRNA capture on beads. Following incubation, beads were removed from the microfluidic device by flushing the beads out into an Eppendorf tube. Following bead removal, reverse transcription and library construction were performed as previously described.^76^

*Fixation and Dehydration of Brain Tumor Stem Cells Near Microvessels for Scanning Electron Microscopy*: For the field emission scanning electron microscopy (FE‐SEM) observation, the plastic chip was fixed and dehydrated with cell‐containing gel 6–10 d postembedding. The transparent gas‐permeable film at the bottom of AIM 3D cell culture chip was carefully delaminated by a tweezer. Fixation and dehydration for SEM preparation was conducted using the same process described in our previous paper.^77^ In brief, the samples were first fixed with 4% paraformaldehyde for 20 min at room temperature and then 2.5% glutaraldehyde for 1 h at 4 °C. Secondary fixing was followed with 1% osmium tetroxide for 1 h at 4 °C. Then, fixed cells were dehydrated by using a grade series of ethanol concentrations (25%, 50%, 75%, and 95%) and followed by final dehydration with 100% ethanol twice for 10 min at 4 °C. Subsequently, dehydrated samples were frozen for 3 h at −80 °C, and then air dried for 24 h in a vacuum desiccator. To observe the microfeatures using FE‐SEM, the samples were sputter‐coated with a layer of iridium (≈12 nm) as a conductive surface layer. An FE‐SEM (SU‐70, Hitachi) was used for observation.

*Data Analysis: Tumor Morphology and Localization Analysis*: The morphology of BTSCs were analyzed with NIS‐Elements software and ImageJ. From day 4 to day 6, representative images of each patient BTSCs with GFP‐HUVECs were selected for analysis. Each raw image of membrane‐labeled fluorescent BTSCs (Data type: TIFF, size: 1392 × 1940 pixels^2^, about 0.12 mm^2^) was binarized by the auto threshold tool and manually segmented through freehand tool. Colocalization correlation of tumor cells and endothelial cells was evaluated by the Pearson's correlation coefficient by the colocalization test tool in Fiji software.^78^, ^79^ The coefficient ranges from −1 to 1: +1 for perfect correlation, 0 for no correlation, and −1 for perfect anticorrelation.

*Permeability Test*: The permeability of microvessels was examined via perfusing 70kDa‐Cy5 dextran (Invitrogen) into the microvessel network. It was measured based on four regions in two independent chips. To avoid border effect, these four regions were mostly in the central region of the gel channel. Dextran was diluted in EGM‐2 medium at a final concentration of 25 µg mL^−1^. All the loading ports were filled with 40 µL medium prior to the test, then 30 µL of dextran was added into one port. A time lapse sequence of the fluorescence intensity was recorded for 30 min, at an interval of 30 s. Once the intravascular fluorescence intensity established equilibrium, typically 5–10 min post injection, permeability was quantified using fluorescence images obtained every 30 s for 2 min. The method described in previous studies was followed to calculate the permeability using the values of average diameter of the lumen (*d*), fluorescence intensity at the intravascular region (*I_0_*), and fluorescence intensity change in the perivascular region (d*I*/d*t*)^32^, ^43^, ^80^

(1) $$P=\frac{d}{4}\times \frac{1}{I_{0}}\times \frac{dI}{dt}$$

*Cell Motility*: GFP‐HUVECs and Dil‐stained patient‐derived GS5 cells in fibrin gel were seeded in the AIM chip and allowed to grow for 48 h. After premature vessel formation, the plate holder of the chip was mounted on a Nikon Eclipse Ti‐S microscope with a motorized stage (Prior Scientific) and an environment control incubation chamber (Okolab) to maintain 37 °C with 5% CO_2_. Phase contrast and fluorescent images were recorded every 30 min for 20 h using a CCD camera (ANDOR) with a low magnification 4X Fluor objective. Each single cell was manually labeled in the continuous frames for 20 h. Cell motility parameters were assessed via tracking of single tumor cells (*n* = 3, ≈20 cells per cropped region per trial) using Fiji. Motility was defined as the distance traveled in a unit time. Cellular displacement was calculated using the corresponding *x* and *y* coordinates at initial time *t*_0_ and end time *t*_n_. Trajectory was plotted by Matlab (R2017a, MathWorks)

(2) $$\mathrm{Displacement}=\sqrt{{\left(x_{n}-x_{0}\right)}^{2}+{\left(y_{n}-y_{0}\right)}^{2}}$$

*Finite Element Simulation (FEM) Analysis*: COMSOL Multiphysics (Version 5.0, COMSOL) software was used to perform finite element analysis on the AIM Biotech microfluidic device set up. 2D fluorescent images of microvessels formed in the AIM Biotech devices were created in COMSOL and simulations were performed to mimic the microenvironment the vessels and cells were experiencing. A whole chip scan image of GBM6 in the microenvironment of green fluorescent HUVEC microvessels (Day 4) was first bianarized using ImageJ. Then, using WinTopo Raster to Vector Conversion Software (v1.76, SoftSoft Ltd.), the black and white BMP images were converted to vectorized images which could be imported into COMSOL. To successfully vectorize the images in WinTopo, various image processing techniques such as erosion, despeckle, and prune were used to create an acceptable image that could be processed using edge detection. The vector file was then imported into COMSOL and used to reconstruct the microvessel environment. The free and porous flow module was applied to simulate for media and fibrin respectively. Media flow with a density of 1020 kg m^−3^ and viscosity of 0.8 cP was governed by the Navier–Stokes and continuity equations for laminar flow.^81^, ^82^ The fibrin was modeled as a porous matrix with a porosity of 0.99 and a permeability of 1.5 × 10^−13^ m^2^ and was simulated using the Brinkman equation.^83^ The geometry was scaled and meshed in COMSOL with a minimum element size of 0.09 mm and a maximum element size of 0.6 mm. For initial conditions, a pressure gradient of 10 Pa from the top to bottom channels was used to represent a column height difference of 1 mm. In the simulation, two inlets were on top and two outlets were on the bottom by assumption. Hydrostatic pressure from a column height of 1 mm representing 10 Pa was used as the inlet pressure while the outlet pressure was set to 0 Pa.

*Single Cell mRNA Sequencing Analysis*: In total, we sequenced 26 027 single cells by using 75 bp pair‐end reads on a HiSeq2500 instrument (Illumina) in HighOutput Mode V4. Raw reads were preprocessed for cell barcodes and UMIs, and then aligned to the human genome(hg19) using STAR v2.5.2b as described in Dropseq method.^84^ Digital expression matrix was generated for the cells with over 10 000 reads per cell.

The Seurat package (V2.3.0) in R (V3.4.1) was applied to identify differentially expressed genes among 26 027 single cells from nine different GBM patients and one GBM cell reference (GS5).^85^ Cells were considered in the analysis only if they met the following quality control criteria: 1) expression of more than 1000 genes and fewer than 5000 genes; 2) low expression of mitochondrial genes (<10% of total counts in a cell). After filtering, 24 120 genes in 21 750 cells were left for clustering analysis. Genes that were differentially expressed in each cluster were identified using the Seurat function FindMarkers, which returned the gene names, average log fold‐change, and adjusted p‐value for genes enriched in each cluster. Unsupervised clustering in principal component analysis (PCA) was performed with 30 statistically significant principal components that were identified from the top 1000 highly variable genes from all the samples. We then projected single cells onto a two‐dimensional map using tSNE to discover interpatient heterogeneity.

The Monocle package (V2.6.4) was used to plot single cell pseudotime trajectories to discover the behavioral similarity and transitions.^64^, ^65^ We use the proneural, mesenchymal, and classical subtype genes identified before to perform the semi‐supervised analysis.^58^ Monocle looked for variable genes and augmented the markers when construct the clustering and ordering of the cells. DDRTree algorithm was used to visualize the pseudotime trajectory in the reduced dimensional space. Plot_genes_branched_heatmap module was applied to plot out the genes (qval<1e‐300) that had similar expression profile on a branch.

*Statistics*: Results were shown as mean ± standard deviation. Student *t*‐test was used to assess the comparisons between the groups in Figure 2. Statistical analysis (one‐way ANOVA) demonstrated significant difference among three groups of these measurements in Figure 3d,e. Statistical significance was assumed for *p* < 0.05, unless otherwise specified. In Figure 5e, a multivariate linear model in JMP (Version 13.0) was performed to show that four predictor variables (gene expression of PDGFRA, C1GALT1, THY1, and MKI67) correlate with colocalization coefficient. The *p*‐values (Table S4, Supporting Information) were calculated based on a multivariate linear mixed model with patient level effect adjusted in R(V3.4.1). Akaike information criterion (AIC) and Bayesian information criterion (BIC) were utilized for model selection. All tests were performed with Prism (Version 7.0, GraphPad Software), JMP (Version 13.0, SAS Institute), or R (V3.4.1).

*Sequencing Data Availability*: The single‐cell RNA‐seq data is available in the Gene Expression Omnibus (GEO) under accession GSE125587.

## Conflict of Interest

Rong Fan is on the Scientific Advisory Boards of IsoPlexis, Bio‐Techne, and Singleron Biotechnologies with financial interest.

## Supporting information

Supplementary

Supplementary

Supplementary

Supplementary
