# Supplementary material for: Ex vivo Dynamics of Human Glioblastoma Cells in a Microvasculature‐on‐a‐Chip System Correlates with Tumor Heterogeneity and Subtypes
Source: Adv Sci (Weinh). 2019 Feb 10;6(8):1801531. doi: 10.1002/advs.201801531 (PMC6468969; doi:10.1002/advs.201801531)
Supplement: Supplementary file 1 — Supplementary [file ADVS-6-1801531-s002.pdf]

Copyright WILEY-VCH Verlag GmbH & Co. KGaA, 69469 Weinheim, Germany, 2019.

ADVANCED SCIENCE
Open Access

Supporting Information

for *Adv. Sci.,* DOI: 10.1002/advs.201801531

Ex vivo Dynamics of Human Glioblastoma Cells in a Microvasculature-on-a-Chip System Correlates with Tumor Heterogeneity and Subtypes

*Yang Xiao, Dongjoo Kim, Burak Dura, Kerou Zhang, Runchen Yan, Huamin Li, Edward Han, Joshua Ip, Pan Zou, Jun Liu, Ann Tai Chen, Alexander O. Vortmeyer, Jiangbing Zhou, and Rong Fan**

SUPPLEMENTARY INFORMATION

Ex vivo behaviors of human glioblastoma cells in a microvasculature-on-a-chip system correlate with tumor heterogeneity and subtypes

Yang Xiao[1], Dongjoo Kim[1], Burak Dura[1], Kerou Zhang[1], Runchen Yan[2], Huamin Li[3], Edward Han[1], Joshua Ip[1], Pan Zou[4], Jun Liu[4], Ann Tai Chen[1], Alexander O. Vortmeyer[5], Jiangbing Zhou[1,4,6], and Rong Fan[1,6] *

[1]Department of Biomedical Engineering, Yale University, New Haven, CT 06520, USA

[2]School of Computer Science, Carnegie Mellon University, Pittsburgh, PA 15213, USA

[3]Applied Math Program, Yale University, New Haven, CT 06520, USA

[4]Department of Neurosurgery, Yale School of Medicine, New Haven, CT 06520, USA

[5]Department of Pathology, Indiana University Health Pathology Laboratory, Indianapolis, IN 46202, USA

[6]Yale Comprehensive Cancer Center, New Haven, CT 06520, USA

* To whom all correspondence should be addressed R.F. (Email: rong.fan@yale.edu)

Contents

Supplementary Figures

Supplementary Figure 1. Rapid formation of HUVEC-only microvasculature on-chip in 4 days.

Supplementary Figure 2. Representative SEM images showed 3D geometry of the tumor cells and vessel network.

Supplementary Figure 3. Cell trajectory (20Hr period) plots drawn in the same scale.

Supplementary Figure 4. Additional simulation results in COMSOL Multiphysics.

Supplementary Figure 5. Unsupervised clustering of single cell mRNA-seq data.

Supplementary Figure 6. Gene markers expression states in the pseudo-time axis.

Supplementary Figure 7. Relative gene expression related to stem cell function and angiogenesis pathway.

Supplementary Tables

Supplementary Table 1. Single Cell RNA-seq Sample Information

Supplementary Table 2. ssGSEA of Stem Cells Analysis

Supplementary Table 3. ssGSEA of GBM Subtype Characterization

Supplementary Table 4. Test Statistics of the Multivariate Mixed Linear Model

Supplementary Table 5. Cell Lines

Supplementary Table 6. Antibodies

Supplementary Table 7. Chemicals and Materials

Supplementary Table 8. Sing Cell mRNA Sequencing

Supplementary Table 9. Software and Algorithms

Supplementary Movies

Supplementary Movie 1. Particle perfusion showing anastomosis of the microvascular network.

Supplementary Movie 2. Confocal reconstruction of 3D BTSCs in the niche.

Supplementary Movie 3. BTSC dynamics in the perivascular niche over 20 hours.

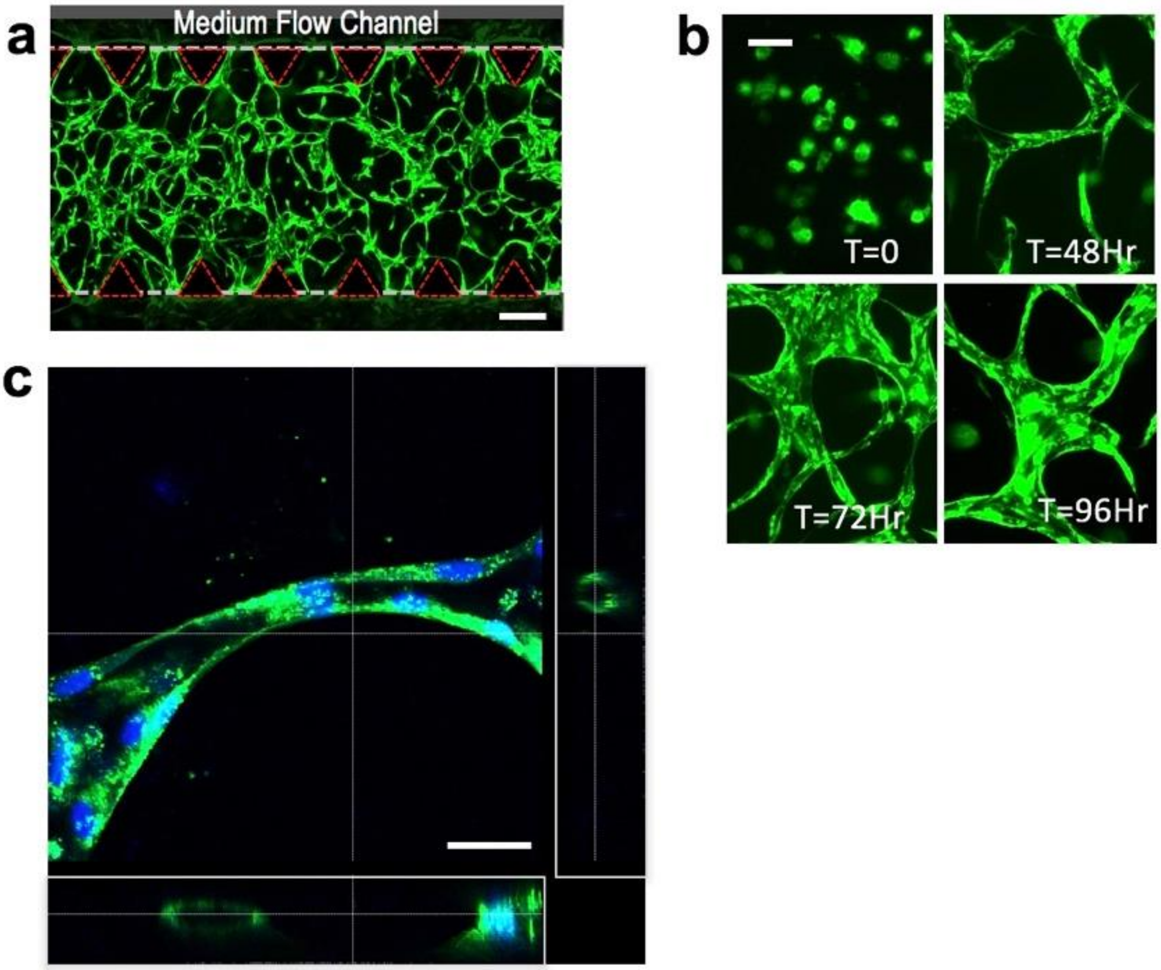

Supplementary Figure 1 Rapid formation of HUVEC-only microvasculature on-chip in 4 days. (**a**) Rapid microvasculature growth 96 hours post-cell loading in fibrin gel with a cocktail of growth factors. Scale bar: 150μm. (**b**) Representative time course of the microvessel formation over 4 days. Scale bar: 15μm. Green: GFP-HUVEC. (**c**) Confocal image of the newly formed micro-vessel (Day 3) confirmed the hollow lumen. Green: GFP-HUVEC. Scale Bar: 30μm.

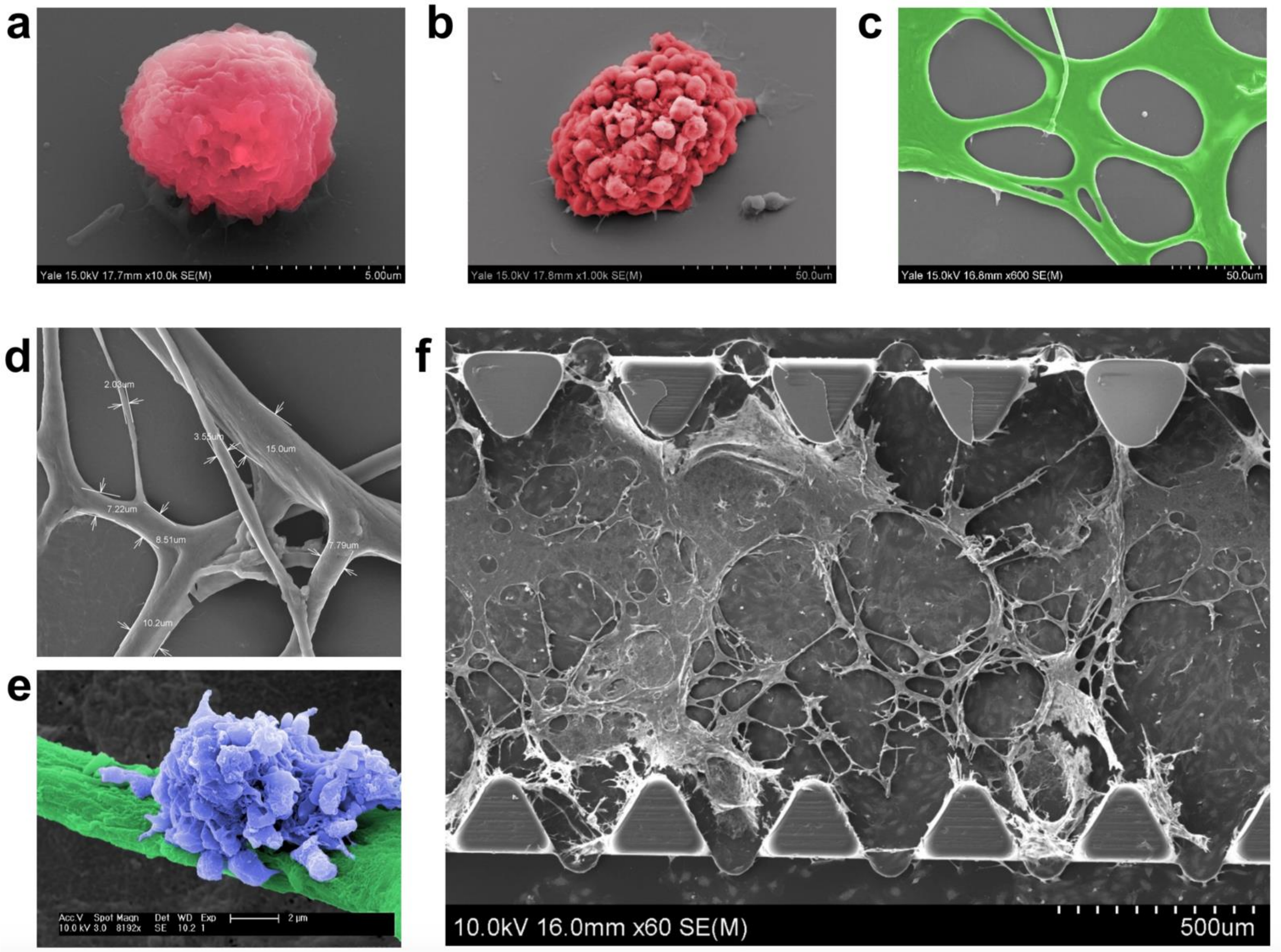

Supplementary Figure 2 Representative SEM images showed 3D geometry of the tumor cells and vessel network. (**a**)Pseudo-color SEM image of a typical GS5 cell. (**b**)Pseudo-color SEM image of a typical GS5 neurosphere. (**c**)Pseudo-color SEM image of a HUVEC-only vessel network. (**d**) SEM image showing the architecture of the microvessels. (**e**)A patient-derived brain tumor stem-like cell (GBM30) attached to the vessel wall. (**f**) A large-scale SEM image of the PVN chip.

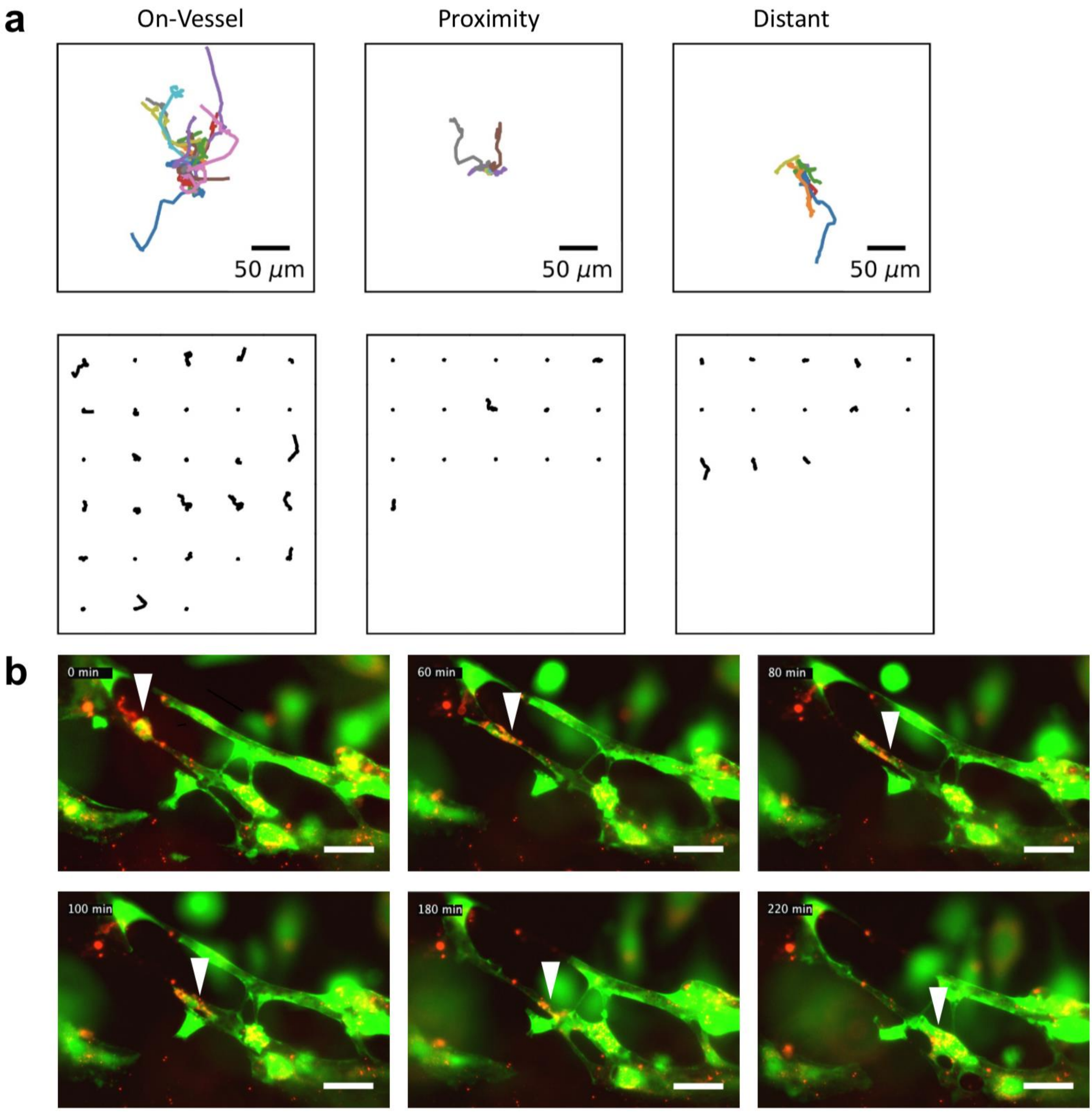

Supplementary Figure 3 GBM migration trajectory on the chip. **(a)** Cell trajectory (20hr period) plots drawn in the same scale, corresponding to Figure 3. On-Vessel (n=28); Proximity (n=16); Distant (n=13). **(b)** A membrane-dyed GBM cell moved along the microvessel at day 20. Scale bar: 50 μm.

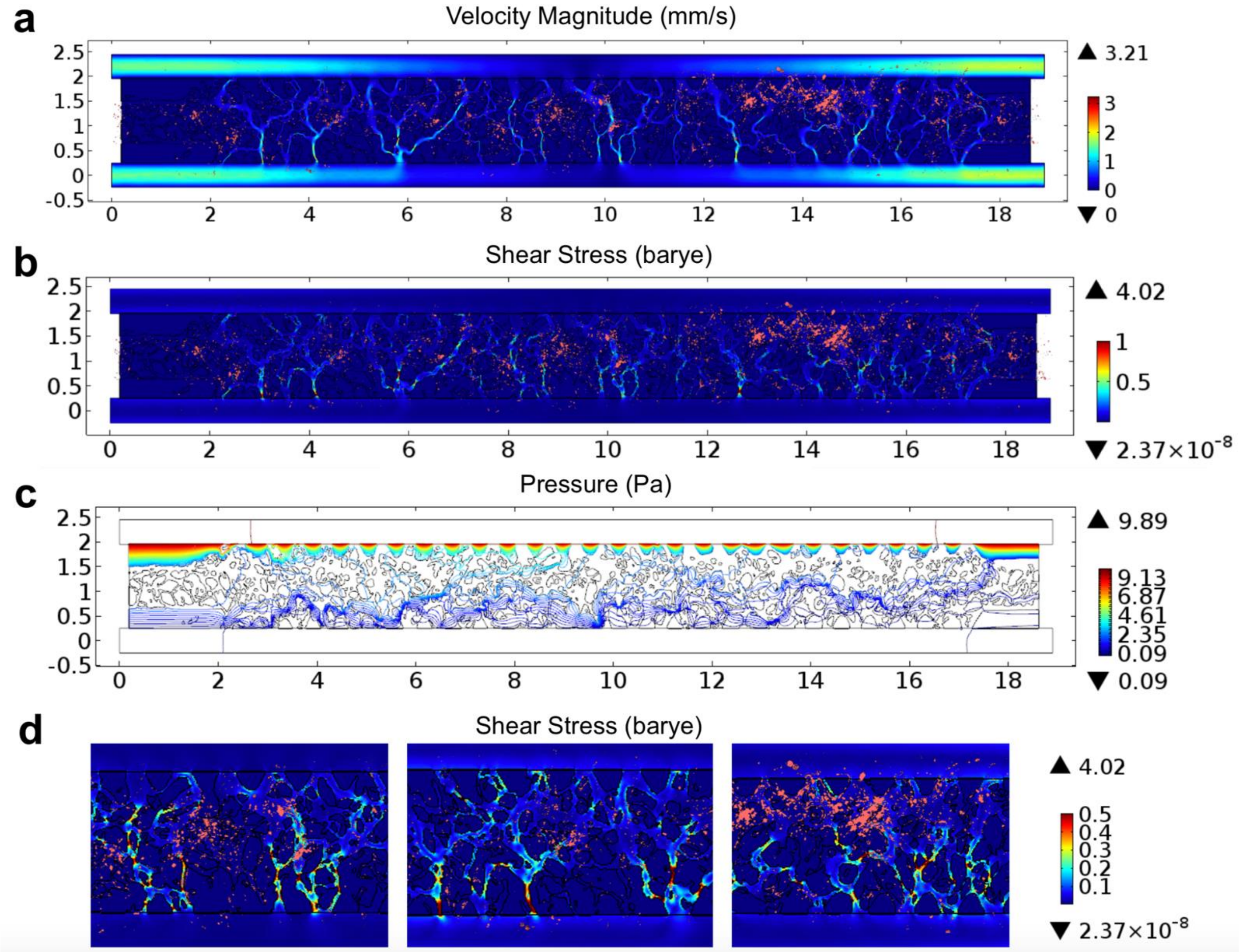

Supplementary Figure 4 Additional simulation results in COMSOL Multiphysics. (**a-b**) Surface plot of velocity profiles and shear stress inside a vascularized AIM Biotech chip. Red glioblastoma cells are overlaid to demonstrate cancer cell localization with respect to microvessels and local flow conditions. (**c**) Contour plot depicting pressure distribution through the vascularized AIM Biotech chip. (**d**) Zoomed in images of 2b showing glioblastoma localization with relation to shear stress. Cancer cells appear to avoid areas of high shear stress and are preferentially located in the upper area of the chip.

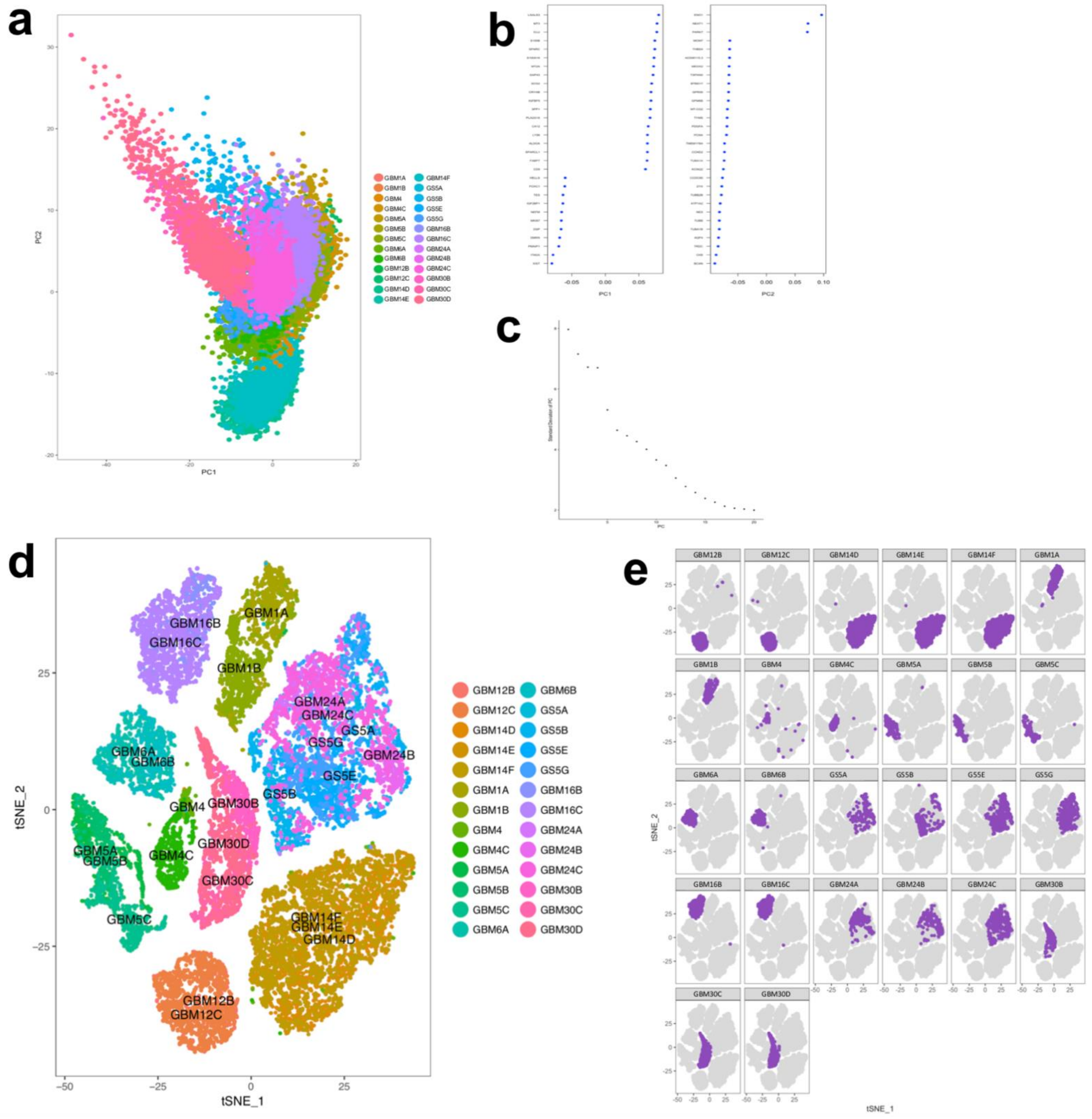

Supplementary Figure 5. **Unsupervised clustering of single cell mRNA-seq data.** (**a**) PCA. (**b**)PCA components. (**c**)Standard deviation of PCA. (**d**)tSNE showing the distribution of 26 batches from 10 patients. (**e**) Monochrome tSNE map of each batch.

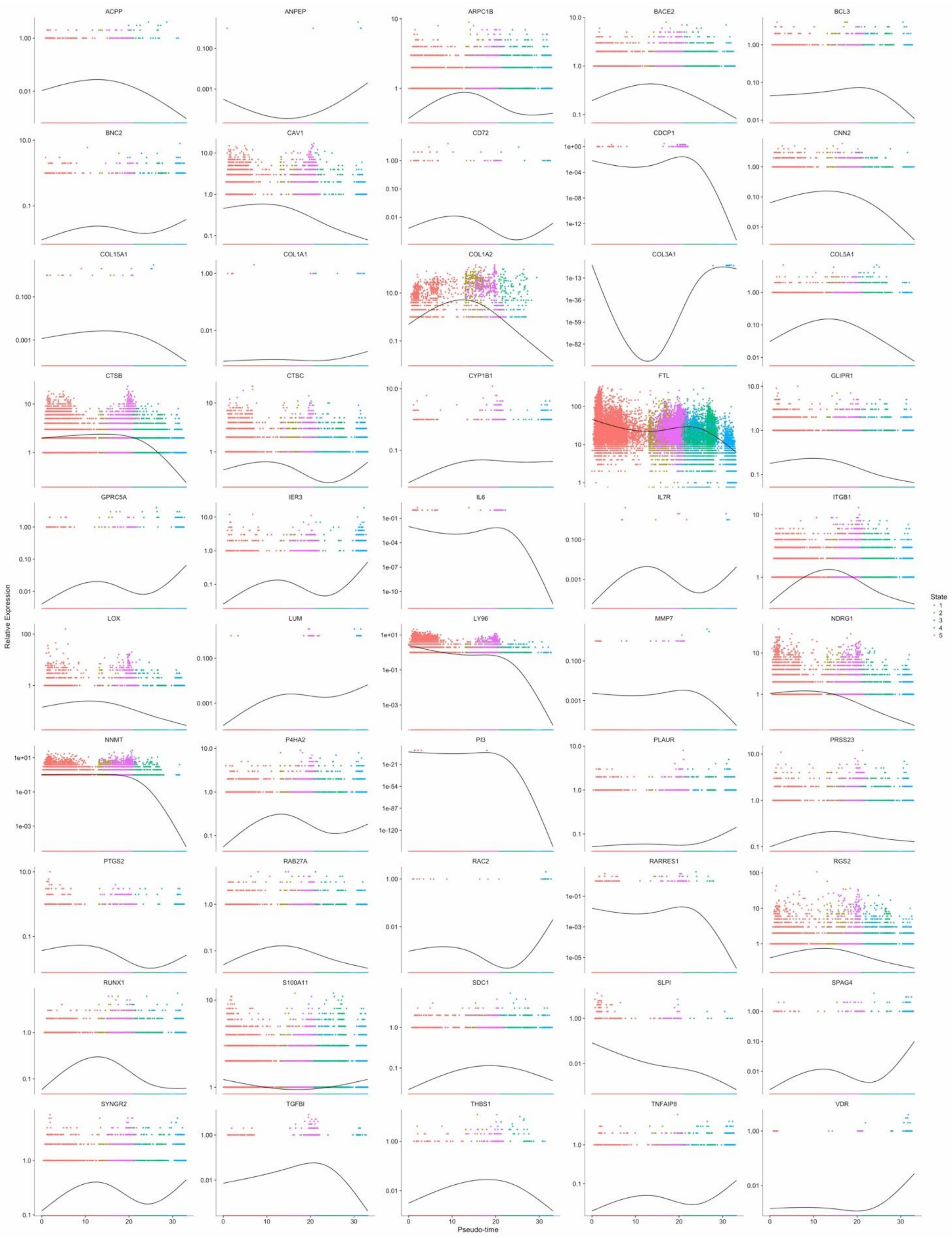

Supplementary Figure 6 Gene markers expression states in the pseudo-time axis.

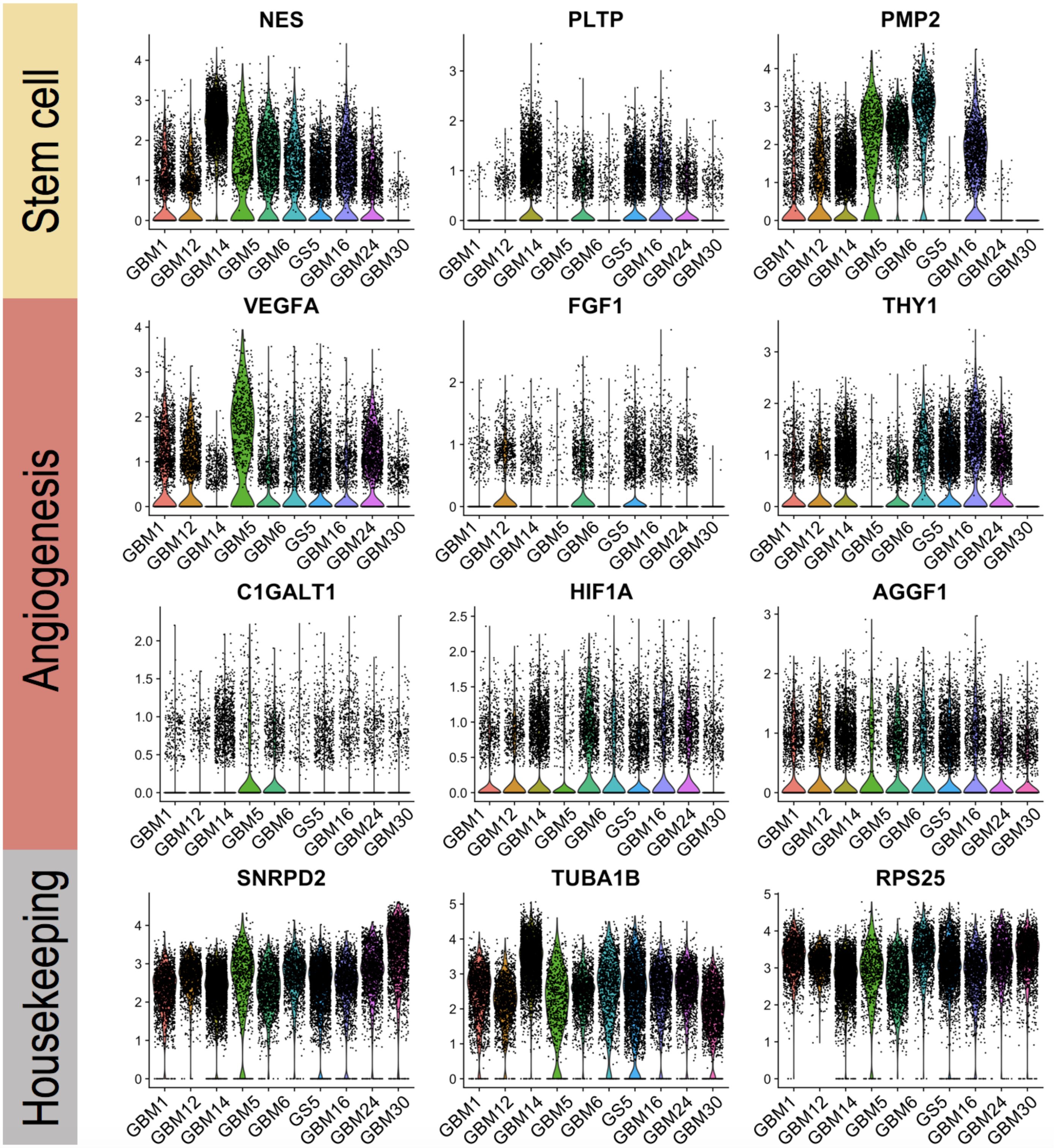

Supplementary Figure 7 Relative gene expression from single cell mRNA-seq related to stem cell function, angiogenesis pathway, and housekeeping.

Supplementary Table 1. Single Cell RNA-seq Sample Information

Name	Number of cells (Pass QC)	Number of cells (Above 10K reads)	Mean reads per cell	Total Number of reads	Median genes per cell	Total genes	Mean UMI per cell	Median UMI per cell	Total UMIs
GBM1A	1057	1183	24466	28942829	2634	22282	10605	7424	12545949
GBM1B	840	883	16957	14973039	2297	20029	8669	7005	7654421
GBM4	152	226	66190	14958911	1740	17254	7077	4408	1599482
GBM4C	654	729	19979	14564842	2005	19972	7119	4899	5189955
GBM5A	622	861	28523	24558038	3621	20679	14369	10010	12372126
GBM5B	540	926	23023	21319488	3626	21067	13842	9874	12818113
GBM5C	562	896	21244	19034257	2417	20545	10053	7611	9007175
GBM6A	1011	1286	34209	43993268	2568	22682	11847	6681	15234651
GBM6B	393	581	44569	25894780	2426	19996	11355	6913	6597182
GBM12B	446	449	13939	6258640	2236	17838	6192	5518	2780261
GBM12C	1248	1276	16168	20630496	2707	21488	9026	7786	11516956
GBM14D	1695	1796	22505	40419467	2563	20331	7858	6398	14113383
GBM14E	1461	1687	24587	41478111	2354	20833	7925	6235	13370240
GBM14F	1435	1583	22676	35896270	2410	20020	8445	6645	13367727
GS5A	316	471	66707	31418884	2816	20540	20174	7989	9501998
GS5B	864	1310	27963	36603973	1811	23500	7414	5258	9711821
GS5E	1389	1825	26661	48656029	2729	21437	11952	9207	21812302
GS5G	843	988	18869	18642521	3018	20023	10149	7956	10027084
PS16B	755	846	23509	19888838	2673	21021	9482	7111	8021547
PS16C	1226	1337	21049	28141902	2368	22171	7426	5517	9929202
PS24A	571	606	17540	10629088	2759	19444	8581	7263	5200067
PS24B	520	527	17761	9360226	2611	18072	10711	8858	5644949
PS24C	871	915	19244	17608113	2620	19943	8653	6745	7917376
PS30B	681	718	17498	12563743	2989	22135	8491	7225	6096274
PS30C	971	1205	17891	21559192	2297	20684	10520	8863	12676876
PS30D	627	917	19304	17701353	2165	20682	9667	7455	8864463
Sum	21750	26027							

Supplementary Table 2. ssGSEA of Stem Cells Analysis

P_{min}<0.05	Stem Cells	Total	Stem Cell %
GBM1	320	1897	16.87%
GBM4	141	806	17.49%
GBM5	281	1724	16.30%
GBM6	201	1404	14.32%
GBM12	250	1694	14.76%
GBM14	553	4591	12.05%
GBM16	245	1981	12.37%
GBM24	172	1962	8.77%
GBM30	132	2279	5.79%
GS5	484	3412	14.19%

Supplementary Table 3. ssGSEA of GBM Subtype Characterization

P_{min}<0.2	CL	MES	NL	PN	CL MES	CL NL	CL PN	MES NL	MES PN	NL PN	CL MES NL	CL MES PN	CL NL PN	MES NL PN	CL MES NL PN	Unidentified	Total
GBM1	293	358	215	325	0	0	9	0	0	1	0	0	0	0	0	696	1897
GBM4	145	145	118	104	2	0	5	0	1	0	0	0	0	0	0	286	806
GBM5	359	263	251	257	0	0	3	0	1	0	0	0	0	0	0	590	1724
GBM6	158	420	140	349	4	1	4	0	1	2	0	0	0	0	0	325	1404
GBM12	289	302	259	266	1	0	3	0	0	0	0	0	0	0	0	574	1694
GBM14	890	834	623	704	2	0	6	0	2	0	0	0	0	0	0	1530	4591
GBM16	329	259	339	332	1	2	4	0	0	1	0	0	0	0	0	714	1981
GBM24	353	244	332	291	0	2	6	0	0	0	0	0	0	0	0	734	1962
GBM30	349	441	319	302	0	0	1	1	0	0	0	0	0	0	0	866	2279
GS5	571	595	472	590	0	0	24	0	2	1	0	0	0	0	0	1157	3412

Supplementary Table 4. Test Statistics of the Multivariate Mixed Linear Model

Covariate	Estimate	95% Confidence Interval	P-value
PDGFRA	0.0180	(0.0104, 0.0257)	<0.0001
MKI67	0.0605	(0.0385, 0.0824)	<0.0001
THY1	0.0382	(0.0139, 0.0625)	0.001
C1GALT1	0.4504	(0.2913, 0.6095)	<0.0001

Supplementary Table 5. Cell Lines

Name	Vendor/Provider	Catalog Number
GFP-HUVEC	Angio-Proteomie	cAP-0001GFP
HUVEC	Yale VBT Core	PRC
RFP-U87	Jiangbing Zhou Lab	N/A
GS5 (GBM patient-derived stem-like cells)	Jiangbing Zhou Lab	N/A

Supplementary Table 6. Antibodies

Name	Vendor	Catalog Number
anti-Collagen IV	Abcam	AB6586
anti-VE-Cadherin	Santa Cruz	SC9989
anti-vWF	Sigma	F3520
Goat anti-rabbit (555nm)	Cell Signaling	4413S
Goat anti-rabbit (647nm)	Cell Signaling	4414S
Goat anti-mouse (488nm)	Cell Signaling	4408S
Goat anti-mouse (555nm)	Cell Signaling	4409S
Goat anti-mouse (647nm)	Cell Signaling	4410S

Supplementary Table 7. Chemicals and Materials

Name	Vendor	Catalog Number
EGM-2 Endothelial Cell Growth Medium-2 Bulletkit	Lonza	CC-3162
VEGF	Peprotech	100-20
EGF	Peprotech	100-15
FGF	Peprotech	100-18B
B27 Supplement	Gibco	17504044
Neural Basal Medium	Invitrogen	10888-022
DMEM/F-12 Medium	Gibco	11320033
Papain	Worthington Biochemical Corp	LK003178
Fibrinogen	Sigma	F8630
Thrombin	Sigma	T7513-100UN
Dil Cell Membrane Dye	Thermo Fisher	V22885
FluoSpheres Polystyrene Microspheres (10 μm)	Invitrogen	F8833
Dextran-647nm	Life Technologies	D22914
DAPI	Invitrogen	D3571
Bovine Serum Albumin (BSA)	Sigma	A9647
DPBS	Life Technologies	A14190144
AIM Biotech 3D Cell Culture Chips	Flexcell	AIMDAX
AIM Biotech Microtiter Plate Holder	Flexcell	AIMHOL

Supplementary Table 8. Sing Cell mRNA Sequencing

Name	Vendor	Catalog Number
Barcoded beads	ChemGenes	5'– Bead–Linker--TTTTTTTAAGCAGTGGTATCAACGCAGAGTACJJJJJJJJJJJJNNNNNNNNTTTTTTTTTTTTTTTTTTTTTTTTTTTTTTT - 3'
Lucigen RNase Inhibitor	Lucigen	30281-1
Fluorinert FC-40	Sigma	F9755-250ml
Maxima-H Minus Reverse Transcriptase	Thermo Fisher	EP0753
dNTP mix	New England Biolabs	N0447S
Template Switch Oligo	IDT	5' - AAGCAGTGGTATCAACGCAGAGTGAATrGrGrG - 3'
20% Ficoll PM-400	Sigma	F5415
Exonuclease I	New England Biolabs	M0293S
Exo I Reaction Buffer	New England Biolabs	B0293S
Kapa HiFi HotStart PCR Mix	Kapa Biosystems	KK2601
SMART PCR Primer	IDT	5' - AAGCAGTGGTATCAACGCAGAGT - 3'
AMPure XP	Beckman Coulter	A63880
Nextera XT DNA Library Preparation Kit	Illumina	FC-131-1096
Nextera N70X oligo	Illumina	FC-131-1001
New-P5-SMART PCR hybrid oligo	IDT	5' - AATGATACGGCGACCACCGAGATCTACACGCCTGTCCGCGGAAGCAGTGGTATCAACGCAGAGT*A*C - 3'
Custom Read 1 Primer	IDT	5' – GCCTGTCCGCGGAAGCAGTGGTATCAACGCAGAGTAC - 3'
DTT	Thermo Fisher	P2325
20% SDS Solution	AmericanBio	AB01922-00500
Alcohol (200 Proof)	AmericanBio	AB00515-00500
Tris - 2M	AmericanBio	AB14116-01000
TWEEN-20	AmericanBio	AB02038-00500
20X SSC Buffer	AmericanBio	AB13156-01000
Water, Nuclease Free, ULTRA PURE	AmericanBio	AB02128-00500
EDTA	AmericanBio	AB00502-01000
PDMS-RTV615	MOMENTIVE	38079

Supplementary Table 9. Software and Algorithms

Name	Reference	Source
NIS-Elements -V4.2	Nikon Instruments	N/A
Huygens Professional	Scientific Volume Imaging	N/A
Prism (v7.0)	GraphPad Software	N/A
JMP (v13.0)	SAS Institute	N/A
COMSOL Multiphysics (v5.0)	COMSOL, Inc.	N/A
Matlab (R2017a)	MathWorks	N/A
Fiji (ImageJ)	Schindelin et al., 2012	http://imagej.net/Fiji
WinTopo (v1.76)	SoftSoft Ltd	http://wintopo.com/
Dropseq_tools (v1.12)	Macosko et al., 2015	http://mccarrolllab.com/dropseq/
STAR (v2.5.2b)	Dobin et al., 2013	https://github.com/alexdobin/STAR
R (v3.4.1)	The R Foundation	https://www.r-project.org
Seurat (v2.3.0)	Satija et al., 2015	http://satijalab.org/seurat/
Monocle (v2.6.4)	Trapnell et al., 2014<br>Qiu et al., 2017	http://cole-trapnell-lab.github.io/monocle-release/
ssGSEA	Wang et al., 2017	https://www.sciencedirect.com/science/article/pii/S1535610817302532#mmc9
Ingenuity Pathway Analysis	Qiagen Bioinformatics	https://www.qiagenbioinformatics.com/products/ingenuity-pathway-analysis/
